# Biomechanical assessment of Kirschner wires integrated with a novel external fixation device for treatment of pediatric supracondylar humeral fracture: a finite element analysis

**DOI:** 10.3389/fbioe.2024.1480298

**Published:** 2024-12-05

**Authors:** Yu-Hsin Lu, Ching-Lung Tai, Wei-Chun Lee, Si-Yao Wang, Chi-Yu Mao, Wen-E. Yang, Chia-Hsieh Chang, Hsuan-Kai Kao

**Affiliations:** ^1^ Department of General Medicine, Chang Gung Memorial Hospital at Linkou, Taoyuan, Taiwan; ^2^ Department of Biomedical Engineering, Chang Gung University, Taoyuan, Taiwan; ^3^ Bone and Joint Research Center, Chang Gung Memorial Hospital at Linkou, Taoyuan, Taiwan; ^4^ Division of Pediatric Orthopaedics, Department of Orthopaedic Surgery, Chang Gung Memorial Hospital at Linkou, Taoyuan, Taiwan; ^5^ College of Medicine, Chang Gung University, Taoyuan, Taiwan

**Keywords:** Kirschner wires, pediatric supracondylar humeral fracture, external fixation device, torsion, bending, finite element analysis

## Abstract

**Background:**

Pediatric supracondylar humeral fractures present considerable surgical challenges due to the difficulty of achieving proper fracture alignment and stable fixation while avoiding injury to the ulnar nerve. This study assesses the biomechanical performance of a novel Kirschner wire (K-wire) fixation device (KFD), designed to enhance stability and reduce complications linked to traditional K-wire configurations.

**Methods:**

Using finite element analysis (FEA), we evaluated four fixation strategies for treatment of pediatric supracondylar humeral simple transverse fractures: crossed pin fixation, crossed pin fixation with KFD, two lateral pin fixation, and two lateral pin fixation with KFD, under various mechanical loads. The analysis focused on the stress and strain experienced by the K-wires at the fracture site during torsional and bending forces.

**Results:**

FEA revealed that the KFD significantly reduced the stress and strain on the K-wires in all configurations. In both crossed pin and two lateral pin fixation methods, the addition of the KFD showed lower stress and strain levels compared to setups without the KFD.

**Conclusion:**

This study demonstrates the potential of the KFD to enhance fracture stability and reduce mechanical stress at the fracture site, suggesting a promising improvement in the treatment of pediatric supracondylar humeral fractures. This innovation may contribute to safer and more reliable outcomes in pediatric orthopedic surgery.

## 1 Introduction

Supracondylar humeral fractures represent the most prevalent type of elbow fractures in children, with their management posing significant clinical challenges (Houshian et al., 2001; Farnsworth et al., 1998). These fractures are commonly classified according to the Gartland system, which categorizes them into three types: Type I (non-displaced), Type II (hinged with intact posterior cortex), and Type III (completely displaced without cortical contact) (Gartland, 1959). Standard treatment protocols recommend closed reduction and internal fixation using percutaneous Kirschner wires (K-wires) for Type II and III fractures (Prashant et al., 2016; Kocher et al., 2007; Skaggs et al., 2004; Gaston et al., 2010; Omid et al., 2008). Despite the widespread application of this method, optimal K-wire configurations remain a subject of debate. While crossed pin fixation is noted for its biomechanical stability, it risks compromising the ulnar nerve (Larson et al., 2006; Marsland and Belkoff, 2014; Lee et al., 2002; Zionts et al., 1994). Alternative method, such as all lateral-entry pin fixation, reduce the risk of ulnar nerve injury and demonstrate comparable clinical outcomes (Kocher et al., 2007; Skaggs et al., 2004; Brauer et al., 2007).

Amidst this backdrop of clinical and biomechanical concerns, the Kirschner wire fixation device (KFD), a novel invention by the senior author Hsuan-Kai Kao, emerges as a potential game-changer. The KFD, detailed in U.S. Patent No. US 10,052,133 B2, offers an innovative approach by securing K-wires in adjustable configurations that potentially enhance stabilization and reduce common complications, such as pin site infection, loss of reduction, loss of fixation associated with traditional methods. In this study, we intent to improve the complication of loss of fixation stability by fixation with K-wires alone. This study utilizes finite element analysis (FEA) to evaluate the biomechanical performance of the KFD, particularly focusing on its efficacy in pediatric supracondylar humeral fractures treated under various K-wire configurations.

## 2 Materials and methods

### 2.1 Generation of the 3-D intact humerus solid model

**FIGURE 1:**
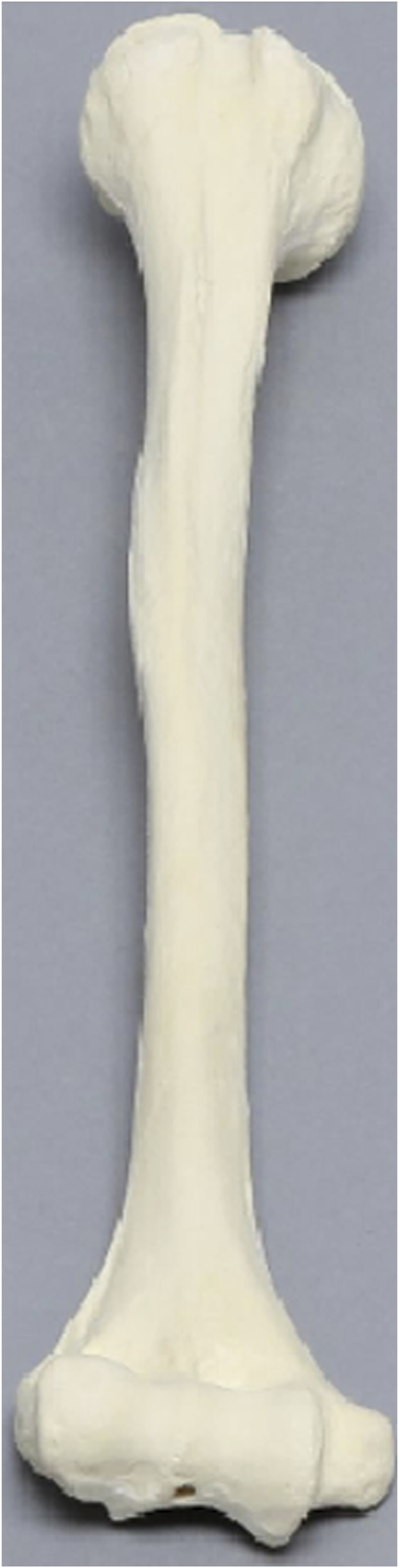
The artificial pediatric humerus bone.

A commercially available synthetic model of pediatic humerus (Model: #1052, Pacific Research Laboratory Inc., Vashon Island, WA, United States) was used to create the finite element (FE) model (Figure 1). Three-dimensional (3D) solid models of a standard humerus were generated using computed tomography (CT) images. The CT images of the intact humerus were captured at 1.25 mm intervals in the transverse plane, starting from the distal end, using a GE Hi-speed scanner (General Electric, Milwaukee, WI, United States). Each CT image had a resolution of 512 by 512 pixels, with a field of view of 320 mm and a pixel size of 0.625 mm/pixel. The obtained cross-sectional images were transferred to an automatic contouring program to delineate the contours between the cortical and cancellous bone. These parallel-stacked contours were then imported into SolidWorks CAD software (SolidWorks Corp., Boston, MA, United States) to reconstruct a 3D solid model of the intact humerus. The solid models of the fixation devices were created based on the dimensions measured from the actual devices (KFD and K-wire).

**FIGURE 2:**
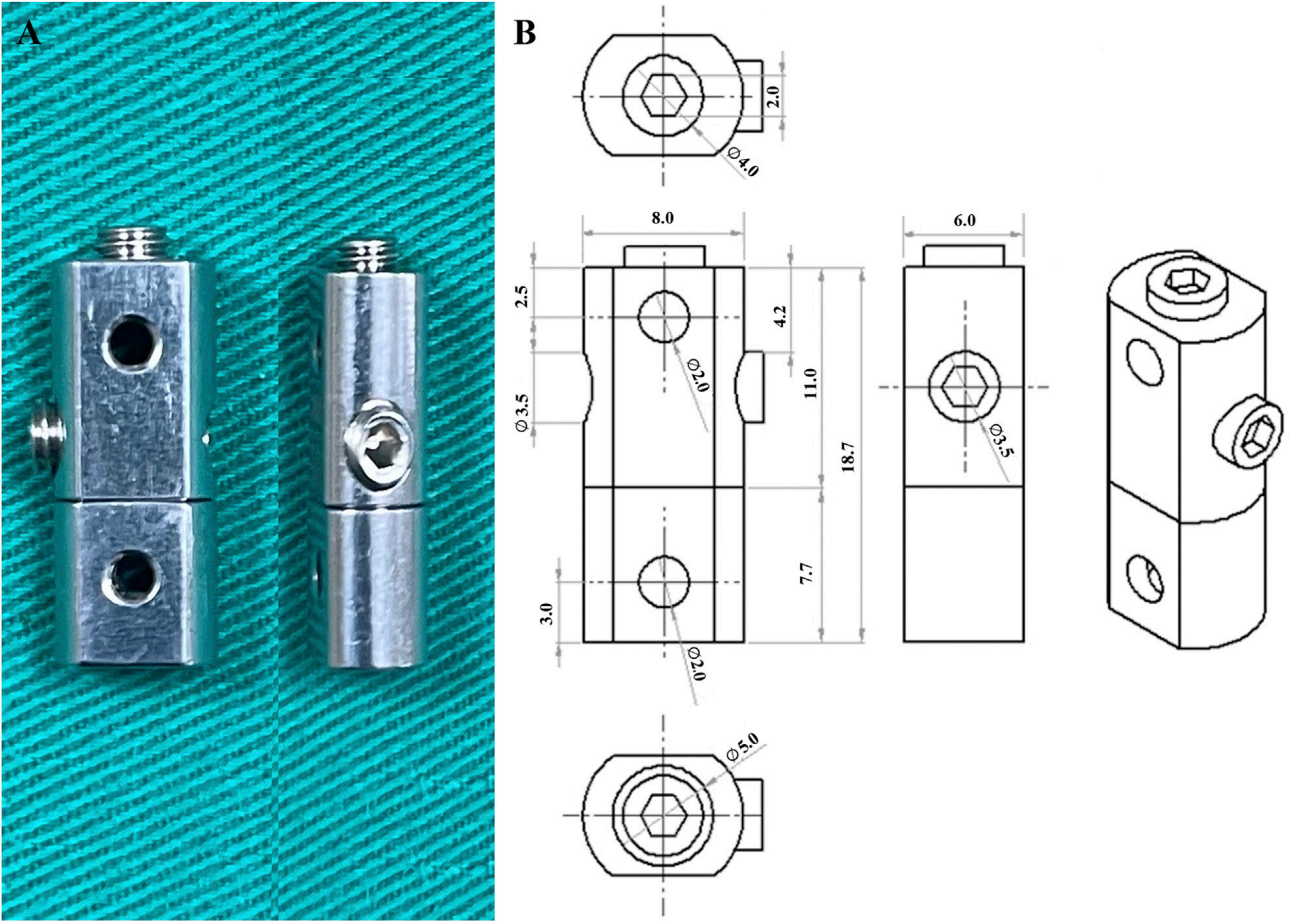
**(A)** Photo and **(B)** schematic of the K-wire fixation device with dimension.

The KFD, constructed from 316L stainless steel, measured 18.7 mm in height 8.0 mm in width, and 6.0 mm in length, with holes having diameter of 2.0 mm (Figure 2). The K-wire was also made of 316L stainless steel and had a diameter of 2.0 mm.

### 2.2 Definition of humerus fixation models

**FIGURE 3:**
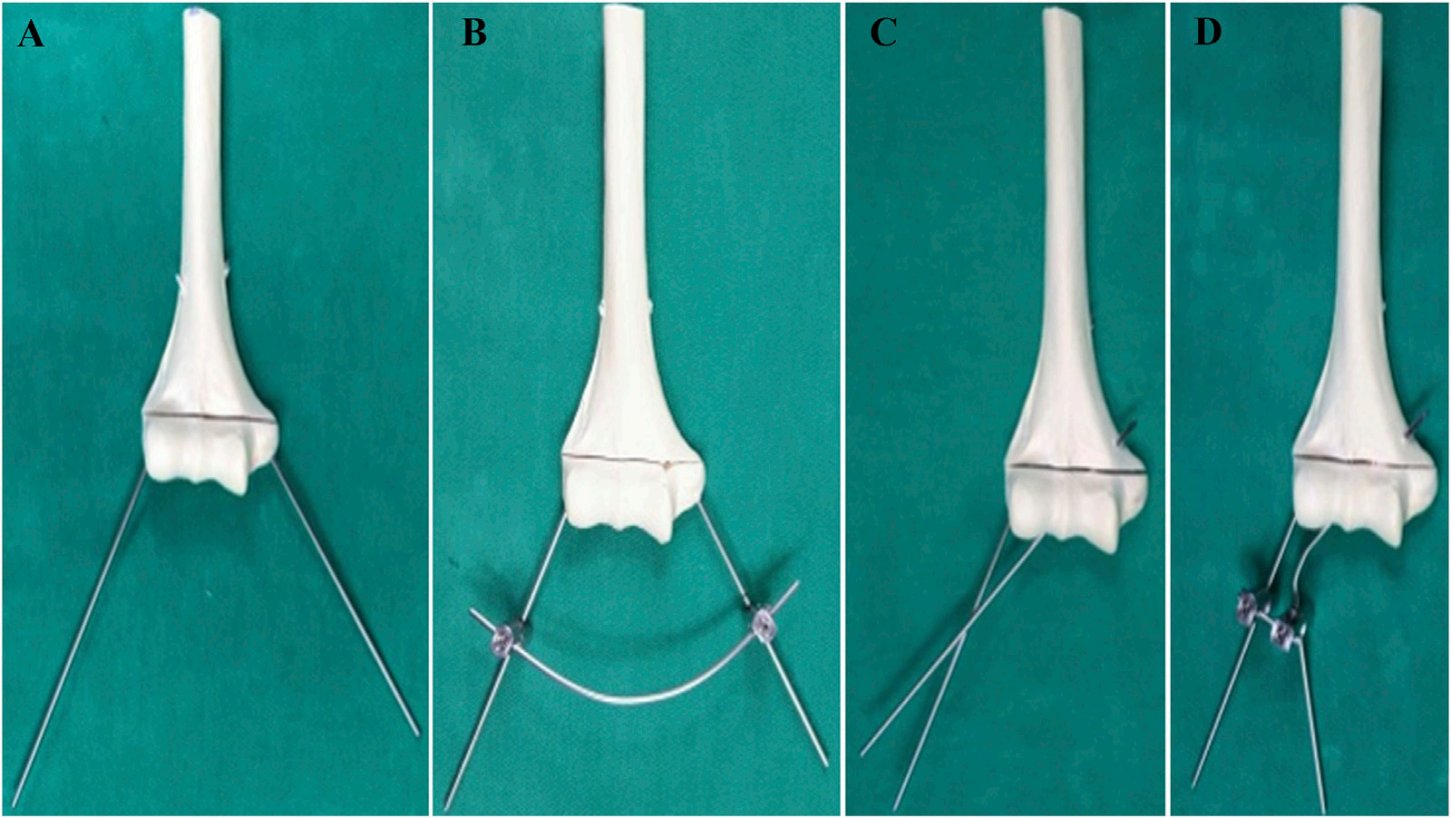
The model of **(A)** crossed pin fixation, **(B)** crossed pin fixation with KFD, **(C)** two lateral pin fixation, **(D)** two lateral pin fixation with KFD.

This study aims to evaluate the effect of the KFD in various K-wire configurations, including crossed pin fixation (C), crossed pin fixation with KFD (C-KFD), two lateral pin fixation (L), and two lateral pin fixation with KFD (L-KFD) (Figure 3).

**FIGURE 4:**
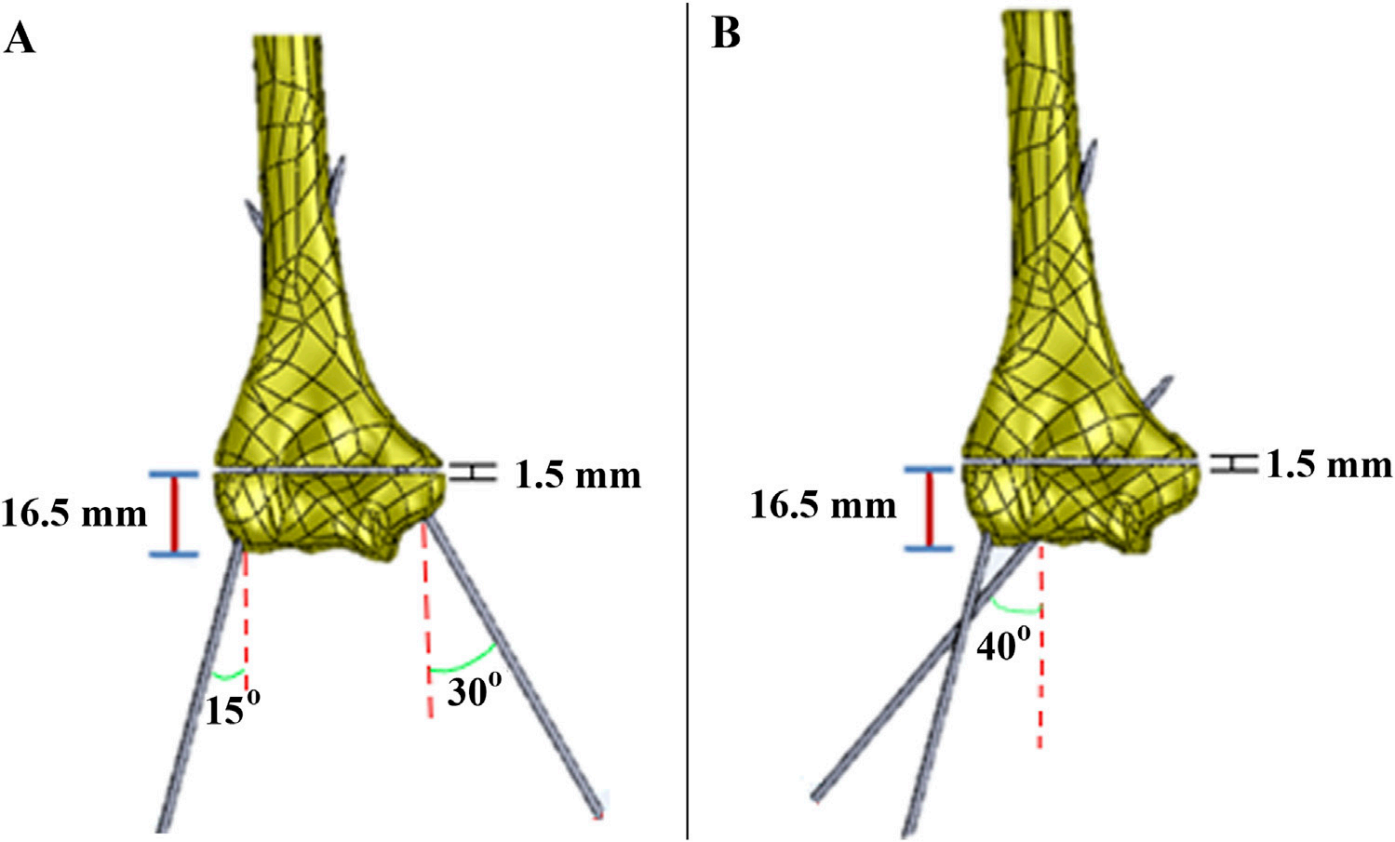
The model of **(A)** crossed pin fixation; and **(B)** two lateral pin fixation.

**FIGURE 5:**
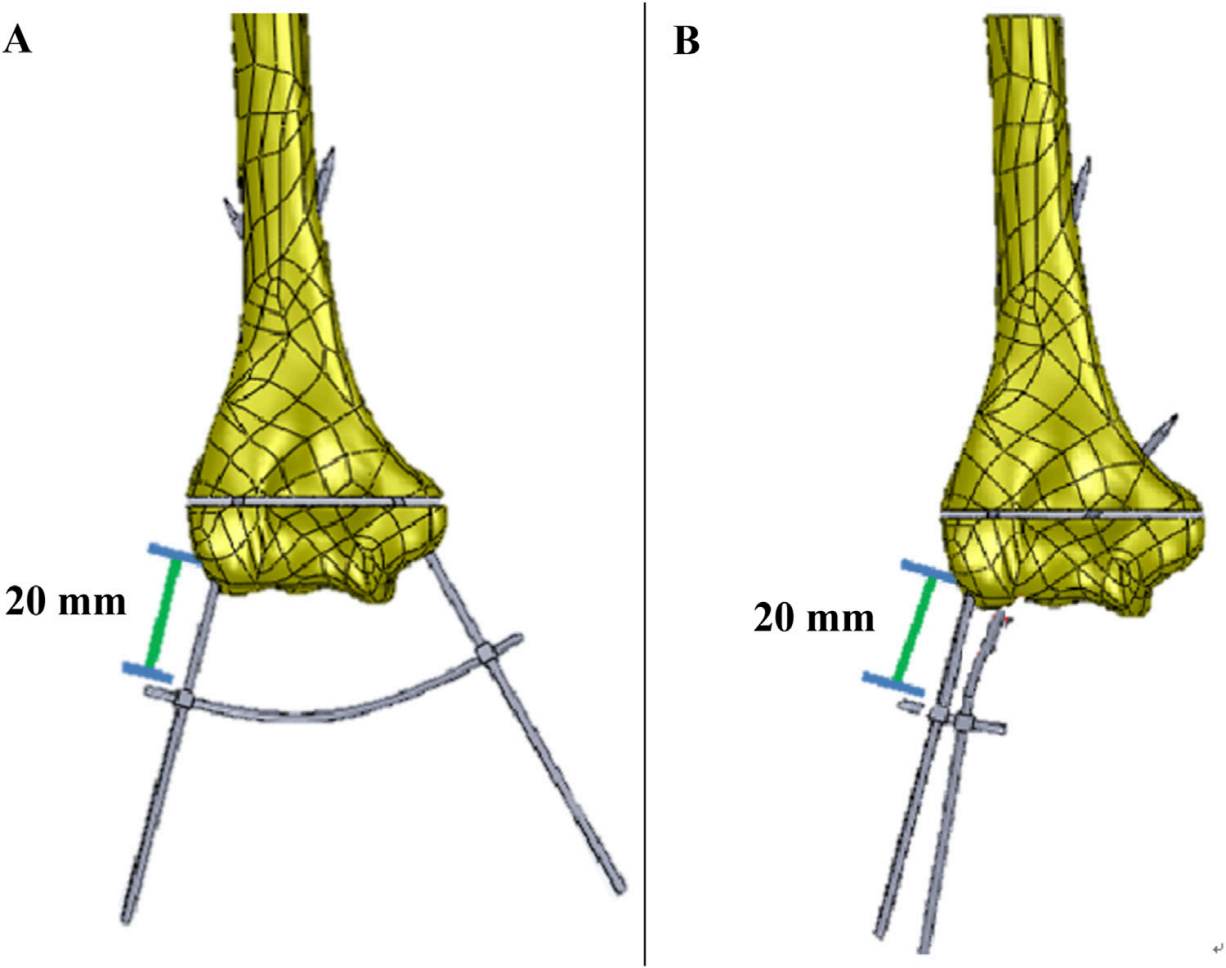
The model of **(A)** crossed pin fixation with KFD, **(B)** two lateral pin fixation with KFD.

The fracture gap was 1.5 mm, located 16.5 mm from the lateral epicondyle. In the crossed pin configuration, one K-wire was inserted at a 30-degree angle and the other at a 15-degree angle. These angles were measured relative to a vertical line drawn perpendicular to the axis connecting the medial and lateral epicondyles. (Figure 4A). In the two lateral pin fixation model, the K-wires were inserted at angles of 15° and 40° with the vertical on the lateral epicondyles and capitulum, respectively (Figure 4B). For both fixation methods, the distance from the bone to the KFD was 20 mm (Figure 5).

### 2.3 Generation of the 3-D finite-element model

Four finite element (FE) models simulating different humerus fixation techniques (C, C-KFD, L, L-KFD) were created by modifying the intact model. The previously established solid models were imported into a commercial finite element software package (Ansys 12.0, Ansys, Inc., Canonsburg, PA, United States). This resulted in a total of four distinct finite element models representing two fixation techniques (C and L) combined with the use of KFD. The element type used for all materials in the FEA model was a 10-node, isoparametric tetrahedral element. All material properties were modeled as a homogeneous linear elastic continuum exhibiting isotropic properties. All contact surfaces between the cortical bone, cancellous bone, and Kirschner wires were modeled as fully bonded.

### 2.4 Loading and boundary conditions

**FIGURE 6:**
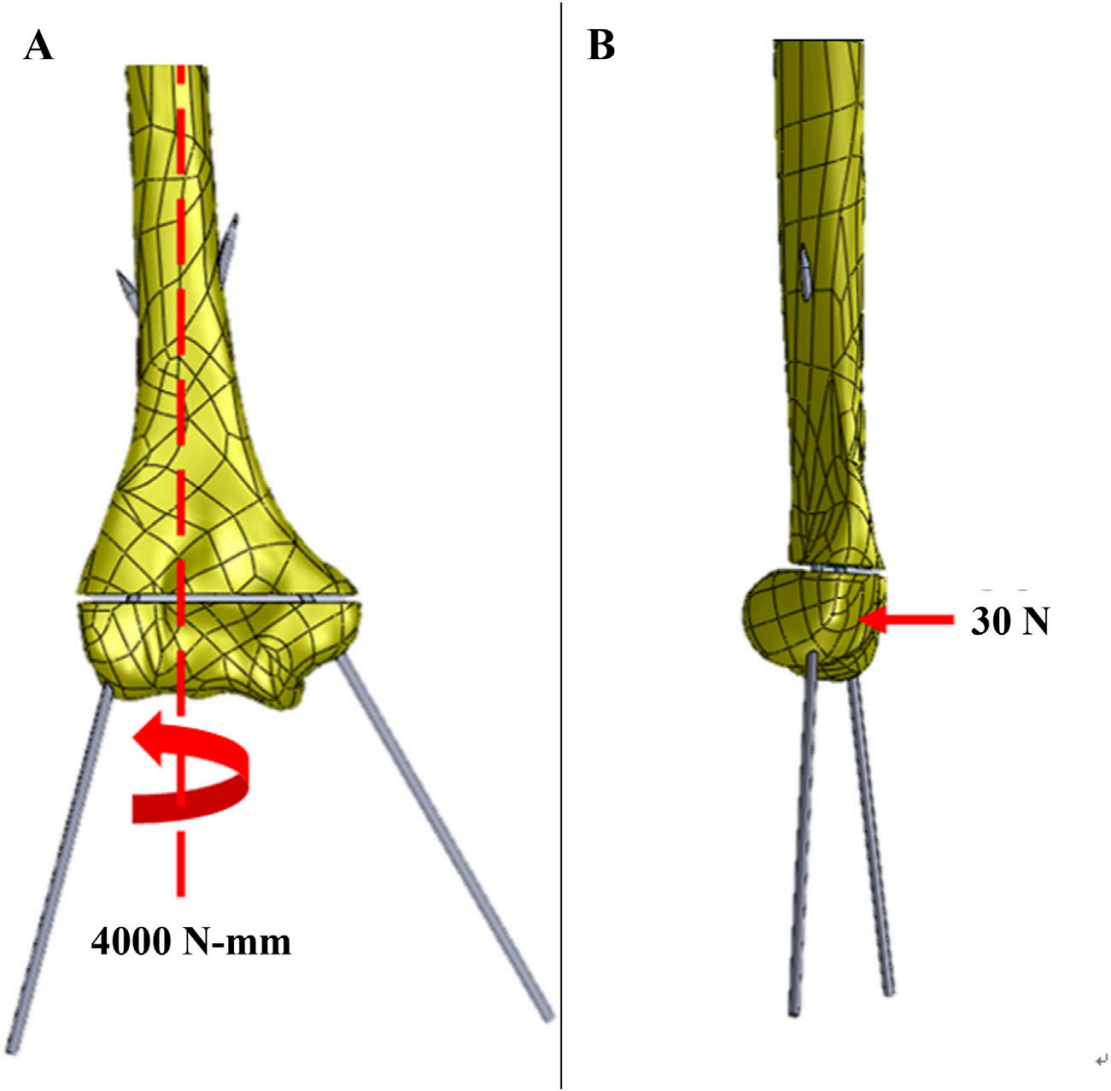
The loading configuration of generated 3-D finite meshes for the **(A)** torsion; and **(B)** bending analysis.

The von Mises stress and strain of the K-wires at the fracture site were compared across all finite element models subjected to torsion and bending external loads. For the torsion analysis, a torque of 4,000 N-mm was applied along the center axis of the humerus shaft. In the bending analysis, a vertical force of 30 N was applied to the humerus shaft at a point 11 mm from the condyle (Figure 6). The Poisson’s ratios used for cortical bone, cancellous bone, and the fixation devices were 0.4, 0.3, and 0.28, respectively, and their moduli of elasticity were 7,000 MPa, 500 MPa, and 205 GPa, respectively (Yu-yong et al., 2008).

### 2.5 Convergence test of the FE models

**FIGURE 7:**
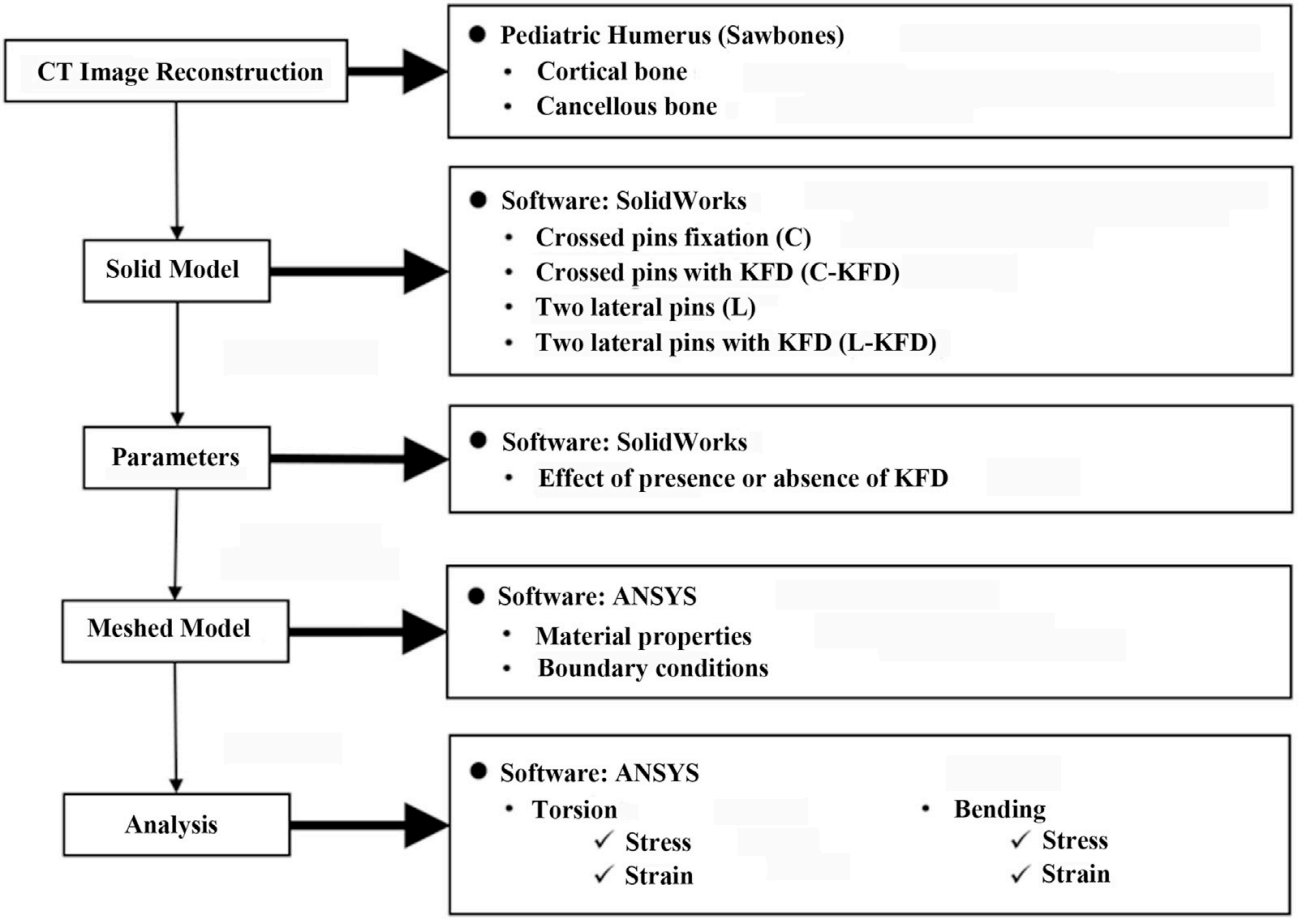
The flow chart of the study.

The convergence of the finite element models in this study was validated by examining the total strain energy of the structure. Six different models with average element lengths of 5, 4.5, 4, 3.5, 3, and 2.5 mm were created, containing 24,014, 25,330, 27,187, 30,551, 35,746, and 43,469 elements, respectively. The total strain energies for these models were 214, 303, 329, 338, 343, and 347 mJ, respectively. The percent differences in total strain energy compared to each nearest model were 29% (5 mm vs. 4.5 mm), 8% (4.5 mm vs. 4 mm), 2.8% (4 mm vs. 3.5 mm), 1.3% (3.5 mm vs. 3 mm), and 1.16% (3 mm vs. 2.5 mm). These percent differences progressively decrease, indicating a converging trend. Based on the convergence test results for these six different mesh refinements and the computational resources required, the model with an average element size of 3.5 mm was selected as the base model for creating the post-operative models. This procedure demonstrated the validity and convergence of the FEA model. The flow chart of the study is shown in Figure 7.

## 3 Results

Stress distribution of K-wires at fracture site: For both crossed pin fixation and two lateral pin fixation, the application of KFD can reduce the stress of the K-wires at the fracture gap under both torsion and bending condition.

**FIGURE 8:**
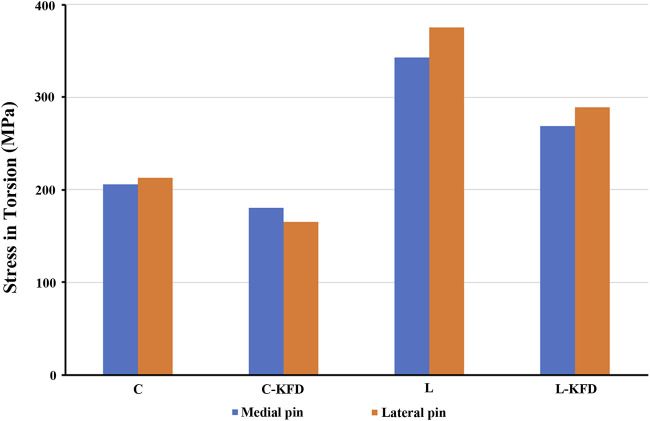
The stress of medial pin and lateral pin at fracture site for various fixation configurations in the torsion analysis.

**FIGURE 9:**
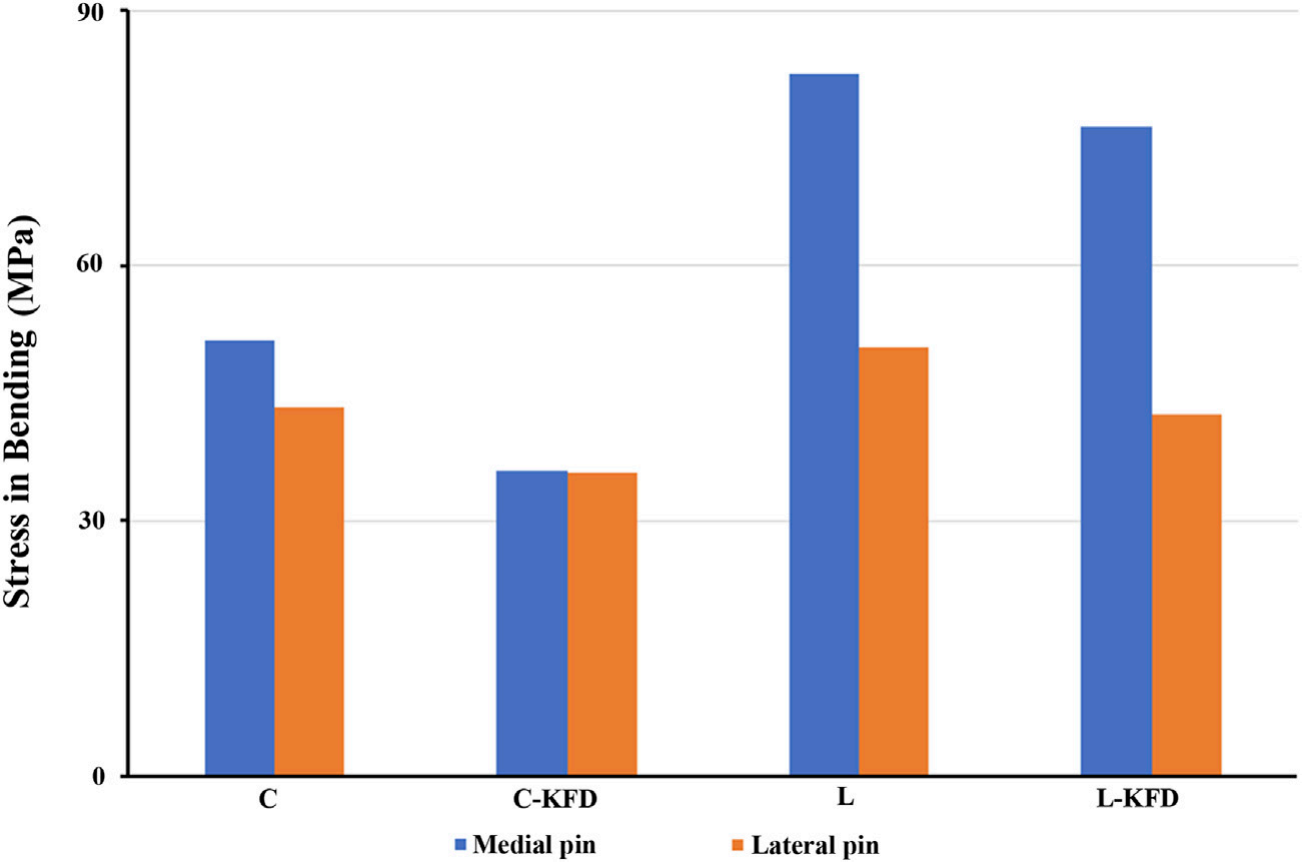
The stress of medial pin and lateral pin at fracture site for various fixation configurations in the bending analysis.

The stress of the medial pin and the lateral pin in torsion and bending analysis are as shown in Figures 8, 9 respectively.

Strain distribution of K-wires at fracture site: For both torsion and bending analysis, the strain of the K-wires in the fracture gap decreases with the KFD applied in both crossed pin fixation and two lateral pin fixation configurations.

**FIGURE 10:**
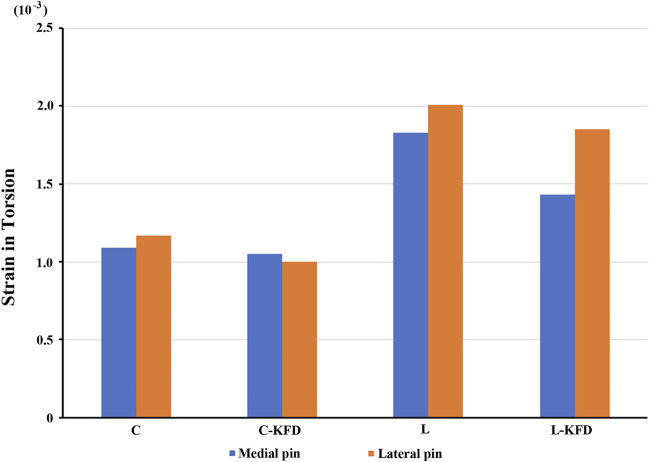
The strain of medial pin and lateral pin at fracture site for various fixation configurations in the torsion analysis.

**FIGURE 11:**
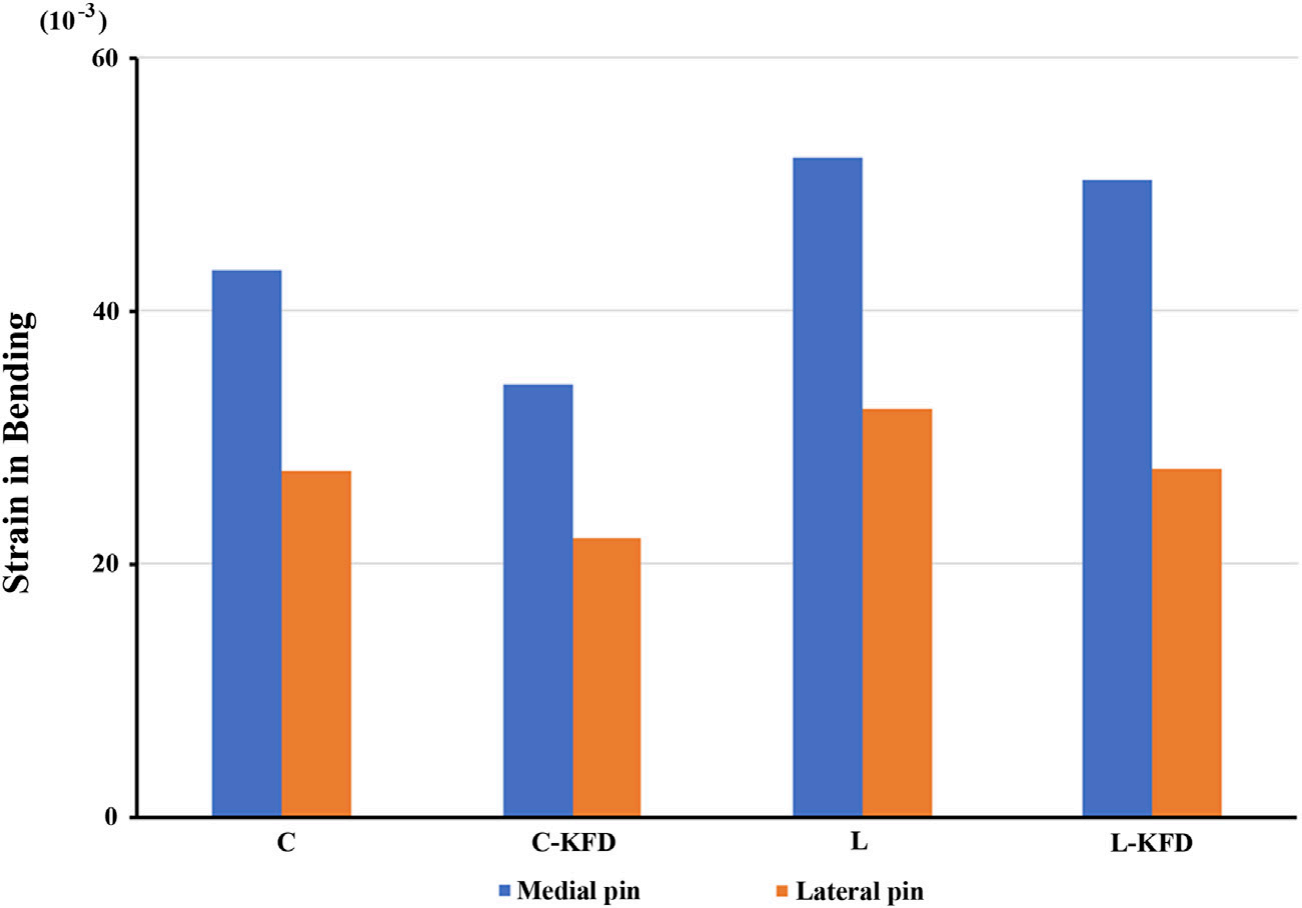
The strain of medial pin and lateral pin at fracture site for various fixation configurations in the bending analysis.

The strain of medial pain and lateral pin at fracture site in torsion and bending analysis are as Figures 10, 11 respectively.

**FIGURE 12:**
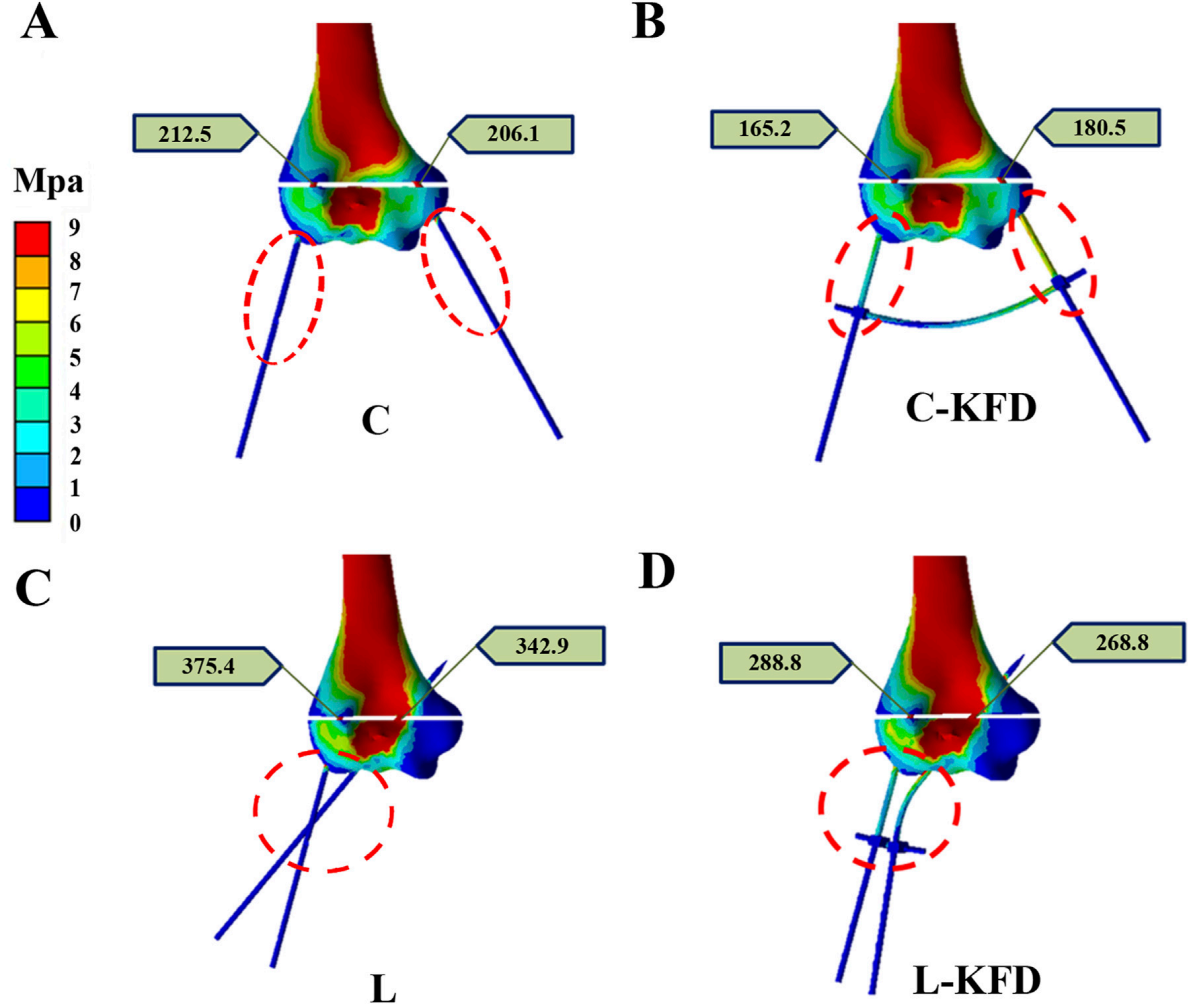
The stress distribution of K-wires of **(A)** crossed pin fixation; **(B)** crossed pin with KFD; **(C)** two lateral pin fixation; and **(D)** two lateral pin with KFD at exposed and fracture sites in the torsion analysis.

Our stress distribution model revealed that KFD enhances fixation stability by altering the stress distribution along the K-wires. This is particularly evident in the torsion analysis, where the application of the KFD results in increased stress on the exposed portion of the K-wire, as illustrated in Figure 12. Conversely, the stress on the K-wire at the fracture site diminishes. The primary factor contributing to this effect is the KFD’s ability to create a stable structure between two K-wires, effectively concentrating stress on the exposed sections of the wires.

## 4 Discussion

To increase the credibility of the result, the biomechanical properties of each material should be as close to reality as possible. We separately set the Young’s modulus and Poisson’s ratio of 316L stainless steel (the material of K-wire and KFD), cortical bone, and cancellous bone. The Young’s modulus of bones in children can vary based on factors such as age, bones from different parts of the body, and the specific area of the bone being examined. Generally, the Young’s modulus of cortical bone in children can range from approximately 5,000–25,000 MPa. Cancellous bone typically has a Young’s modulus ranging from around 10–500 MPa in children (Semaan et al., 2019; Wu et al., 2018). However, these values can vary depending on individual factors. Therefore, in this study the Young’s modulus of cortical bone and cancellous bone are set to be 7,000 MPa and 500 MPa, respectively (Semaan et al., 2019; Wu et al., 2018). The Poisson’s ratio of child bone is also vary based on factors such as age and bone type. Generally, for cortical bone in children, the Poisson’s ratio falls within the range of approximately 0.2–0.4. Cancellous bone typically has a Poisson’s ratio ranging from around 0.15 to 0.3 in children. Thus, in this study the Poisson’s ratio of cortical bone and cancellous bone are set to be 0.4 and 0.3, respectively (Yu-yong et al., 2008; Semaan et al., 2019; Wu et al., 2018; Hoffmeister et al., 2000; Öhman et al., 2011; Watanabe et al., 2000).

External fixation stabilizes fractures by inserting pins or wires into the bone and connecting them to an external frame, allowing adjustable alignment and load distribution, thus reducing stress at the fracture site (Fernando et al., 2021; Hadeed et al., 2024; Fragomen and Rozbruch, 2007; Aronson and Harp, 1992). The addition of KFD can significantly improve postoperative stability in several ways. By connecting the K-wires, the KFD transforms the fixation into a more robust external skeletal system. This configuration reduces both stress and strain on the K-wires at the fracture site during torsional and bending forces. The KFD also cleverly redistributes stress along the wires, concentrating it on the exposed parts while decreasing it at the crucial fracture area. This redistribution creates a more stable overall structure that’s better equipped to resist various types of forces, including compression and shear. The enhanced stability provided by the KFD likely decreases the relative excessive movement between fractured fragments at the fracture site, which is beneficial for bone healing. It also addresses common complications associated with traditional K-wire fixation, such as loss of reduction, pin migration, and loss of fixation. These improvements mean that the fracture is held more securely in place, reducing the risk of fixation failure that might require additional surgery. In essence, the KFD takes the standard K-wire fixation method and enhances its biomechanical performance. By providing a more stable environment for the fracture to heal, it has the potential to improve clinical outcomes in the treatment of pediatric supracondylar humeral fractures.

Our study utilized finite element analysis to evaluate the biomechanical efficacy of a novel KFD in pediatric supracondylar humeral fractures. The results demonstrate significant potential for improving fracture stability and reducing mechanical stress on K-wires. These findings have several important clinical implications: 1). Reduced risk of loss of reduction: By providing a more robust fixation, the KFD may decrease the likelihood of post-operative loss of reduction, a complication that often necessitates revision surgery. 2). Lower rates of pin migration: The decreased stress on K-wires could potentially reduce the risk of pin migration, a common complication that can lead to loss of fixation and compromise patient outcomes. 3). Improved bone healing: The more stable environment created by the KFD may promote better bone healing by limiting excessive movement between fractured segments at the fracture site. In conclusion, our biomechanical analysis suggests that the KFD has the potential to significantly improve the management of pediatric supracondylar humeral fractures. By enhancing stability and reducing stress on K-wires, this novel device may lead to better clinical outcomes, reduced complication rates, and improved patient experiences. However, clinical validation and long-term studies are necessary to fully understand its impact and optimal application in pediatric orthopedic practice.

This study has several limitations. First, the FEA model simplifies the complexity of biological tissues, excluding elements such as muscles, tendons, and nerves. Although this may introduce some error, it enables a more focused investigation of bone and fixation mechanics. Future models could incorporate soft tissue properties to improve the accuracy of FEA results. Second, the interfaces between cortical bone, cancellous bone and K-wires were modeled as “bonded.” While this approach simplifies the simulation, it may introduce biases compared to reality. More realistic modeling of these interfaces, such as accounting for potential loosening, would capture important clinical factors. Future studies could implement frictional contact or cohesive zone models to simulate the bone-wire interface more accurately. Third, the actual loads acting on bone are multidirectional. This study focused on torsion and bending to investigate critical fracture stresses. The KFD technique enhances traditional K-wire fixation by creating semi-triangular structures, potentially increasing overall stability and reducing wire migration. This configuration likely improves the K-wires’ resistance to compression and shear forces. Although this research focused on torsion and bending, future studies that incorporate axial and shear forces would provide a more comprehensive biomechanical evaluation.

## 5 Conclusion

The findings of this study suggest that KFD may improve fracture stability and lessen the mechanical load on K-wires, potentially leading to better outcomes in treating pediatric supracondylar humeral fractures.

## Data Availability

The original contributions presented in the study are included in the article/supplementary material, further inquiries can be directed to the corresponding author.

## References

[B1] Aronson J. Harp J. H. Jr. (1992). Mechanical considerations in using tensioned wires in a transosseous external fixation system. Clin. Orthop. Relat. Res. 280, 23–29. 10.1097/00003086-199207000-00005 1611749

[B2] Brauer C. A. Lee B. M. Bae D. S. Waters P. M. Kocher M. S. (2007). A systematic review of medial and lateral entry pinning versus lateral entry pinning for supracondylar fractures of the humerus. J. Pediatr. Orthop. 27 (2), 181–186. 10.1097/bpo.0b013e3180316cf1 17314643

[B4] Farnsworth C. L. Silva P. D. Mubarak S. J. (1998). Etiology of supracondylar humerus fractures. J. Pediatr. Orthop. 18 (1), 38–42. 10.1097/01241398-199801000-00008 9449099

[B5] Fernando P. L. N. Abeygunawardane A. Wijesinghe P. Dharmaratne P. Silva P. (2021). An engineering review of external fixators. Med. Eng. Phys. 98, 91–103. 10.1016/j.medengphy.2021.11.002 34848044 PMC8660649

[B6] Fragomen A. T. Rozbruch S. R. (2007). The mechanics of external fixation. Hss J. 3 (1), 13–29. 10.1007/s11420-006-9025-0 18751766 PMC2504087

[B7] Gartland J. J. (1959). Management of supracondylar fractures of the humerus in children. Surg. Gynecol. Obstet. 109 (2), 145–154. 13675986

[B8] Gaston R. G. Cates T. B. Devito D. Schmitz M. Schrader T. Busch M. (2010). Medial and lateral pin versus lateral-entry pin fixation for Type 3 supracondylar fractures in children: a prospective, surgeon-randomized study. J. Pediatr. Orthop. 30 (8), 799–806. 10.1097/bpo.0b013e3181f73d59 21102204

[B9] Hadeed A. Werntz R. L. Varacallo M. (2024). “External fixation principles and overview,” in StatPearls treasure Island (FL): StatPearls publishing copyright © 2024 (Tampa, FL: StatPearls Publishing LLC.). 31613474

[B10] Hoffmeister B. Smith S. Handley S. Rho J. (2000). Anisotropy of Young's modulus of human tibial cortical bone. Med. Biol. Eng. Comput. 38, 333–338. 10.1007/bf02347055 10912351

[B11] Houshian S. Mehdi B. Larsen M. S. (2001). The epidemiology of elbow fracture in children: analysis of 355 fractures, with special reference to supracondylar humerus fractures. J. Orthop. Sci. 6 (4), 312–315. 10.1007/s007760100024 11479758

[B14] Kocher M. S. Kasser J. R. Waters P. M. Bae D. Snyder B. D. Hresko M. T. (2007). Lateral entry compared with medial and lateral entry pin fixation for completely displaced supracondylar humeral fractures in children. A randomized clinical trial. J. Bone Jt. Surg. Am. 89 (4), 706–712. 10.2106/00004623-200704000-00002 17403790

[B15] Larson L. Firoozbakhsh K. Passarelli R. Bosch P. (2006). Biomechanical analysis of pinning techniques for pediatric supracondylar humerus fractures. J. Pediatr. Orthop. 26 (5), 573–578. 10.1097/01.bpo.0000230336.26652.1c 16932093

[B17] Lee S. S. Mahar A. T. Miesen D. Newton P. O. (2002). Displaced pediatric supracondylar humerus fractures: biomechanical analysis of percutaneous pinning techniques. J. Pediatr. Orthop. 22 (4), 440–443. 10.1097/01241398-200207000-00005 12131437

[B18] Marsland D. Belkoff S. M. (2014). Biomechanical analysis of posterior intrafocal pin fixation for the pediatric supracondylar humeral fracture. J. Pediatr. Orthop. 34 (1), 40–44. 10.1097/bpo.0b013e31829b2ef7 23812145

[B19] Öhman C. Baleani M. Pani C. Taddei F. Alberghini M. Viceconti M. (2011). Compressive behaviour of child and adult cortical bone. Bone 49 (4), 769–776. 10.1016/j.bone.2011.06.035 21763479

[B20] Omid R. Choi P. D. Skaggs D. L. (2008). Supracondylar humeral fractures in children. J. Bone Jt. Surg. Am. 90 (5), 1121–1132. 10.2106/jbjs.g.01354 18451407

[B21] Prashant K. Lakhotia D. Bhattacharyya T. D. Mahanta A. K. Ravoof A. (2016). A comparative study of two percutaneous pinning techniques (lateral vs medial-lateral) for Gartland type III pediatric supracondylar fracture of the humerus. J. Orthop. Traumatol. 17 (3), 223–229. 10.1007/s10195-016-0410-2 27312248 PMC4999378

[B22] Semaan M. Karam E. Baron C. Pithioux M. (2019). Estimation of the elastic modulus of child cortical bone specimens via microindentation. Connect. Tissue Res. 60 (4), 399–405. 10.1080/03008207.2019.1570170 30646770

[B23] Skaggs D. L. Cluck M. W. Mostofi A. Flynn J. M. Kay R. M. (2004). Lateral-entry pin fixation in the management of supracondylar fractures in children. J. Bone Jt. Surg. Am. 86 (4), 702–707. 10.2106/00004623-200404000-00006 15069133

[B24] Watanabe Y. Shiba N. Matsuo S. Higuchi F. Tagawa Y. Inoue A. (2000). Biomechanical study of the resurfacing hip arthroplasty: finite element analysis of the femoral component. J. Arthroplasty 15 (4), 505–511. 10.1054/arth.2000.1359 10884212

[B25] Wu D. Isaksson P. Ferguson S. J. Persson C. (2018). Young’s modulus of trabecular bone at the tissue level: a review. Acta biomater. 78, 1–12. 10.1016/j.actbio.2018.08.001 30081232

[B26] Yu-yong D. Yun-sheng Y. Xiao-feng X. Yuan-hui D. (2008). Construction and validation of the finite element model of humeral supracondylar fracture in children. J. Clin. Rehabilitative Tissue Eng. Res. 12 (22), 4265–4269.

[B27] Zionts L. E. McKellop H. A. Hathaway R. (1994). Torsional strength of pin configurations used to fix supracondylar fractures of the humerus in children. J. Bone Jt. Surg. Am. 76 (2), 253–256. 10.2106/00004623-199402000-00013 8113261

